# Supplementary material for: Maternal Vaccination as an Integral Part of Life-Course Immunization: A Scoping Review of Uptake, Barriers, Facilitators, and Vaccine Hesitancy for Antenatal Vaccination in Ireland
Source: Vaccines (Basel). 2025 May 23;13(6):557. doi: 10.3390/vaccines13060557 (PMC12197377; doi:10.3390/vaccines13060557)
Supplement: Supplementary file 1 [file vaccines-13-00557-s001.zip › vaccines-3347433-supplementary/vaccines-3347433-supplementary/National immunisation schedule of Ireland- Vaccines- Supplementary file- April 2025.pdf]

Immunisation Schedule

When	Vaccination	Where	
2 months	6 in 1<br>MenB<br>PCV<br>Rotavirus	GP	Visit 1
4 months	6 in 1<br>MenB<br>Rotavirus	GP	Visit 2
6 months	6 in 1<br>PCV	GP	Visit 3
	No Rotavirus vaccine on or after 8 months 0 days		
12 months	MMR<br>MenB<br>Chickenpox	GP	Visit 4
13 months	6 in 1<br>MenC<br>PCV	GP	Visit 5<br>Five GP visits complete
Junior Infants	4 in 1<br>MMR	Primary School	4 in 1 & MMR vaccines
1st year	HPV<br>Tdap<br>MenACWY	Secondary School	HPV vaccine, Tdap vaccine, Men ACWY vaccine
Pregnancy	Tdap<br>(from 16 weeks)	GP	Tdap vaccine
Recommended Groups	Flu<br>(Nasal vaccine 2-17 yrs, injection for everyone else)	GP<br>Pharmacy<br>HSE Vaccinator	
At - risk	PPV	GP	
6+ months	COVID-19	GP<br>Pharmacy<br>Vaccination Centres	

PROTECT PREVENT IMMUNISE

Vaccine Abbreviations

6 in 1 - Diphtheria, haemophilus influenzae type b (Hib), hepatitis B, acellular pertussis, inactivated polio, tetanus vaccine
MenB - Meningococcal B recombinant vaccine
Rotavirus - Rotavirus oral vaccine
PCV - Pneumococcal conjugate vaccine
MenC - Meningococcal C conjugate vaccine
MMR - Measles, mumps, rubella vaccine
Chickenpox - Varicella vaccine
4 in 1 - Diphtheria, tetanus, pertussis, polio vaccine
HPV - Human papillomavirus vaccine
Tdap - Low dose diphtheria, tetanus and acellular pertussis vaccine
MenACWY - Meningococcal A, C, W, Y conjugate vaccine
Flu - Influenza vaccine
PPV - Pneumococcal polysaccharide vaccine
COVID-19 - COVID-19 Vaccine

October 2024
