# Supplementary material for: Maternal Vaccination as an Integral Part of Life-Course Immunization: A Scoping Review of Uptake, Barriers, Facilitators, and Vaccine Hesitancy for Antenatal Vaccination in Ireland
Source: Vaccines (Basel). 2025 May 23;13(6):557. doi: 10.3390/vaccines13060557 (PMC12197377; doi:10.3390/vaccines13060557)
Supplement: Supplementary file 1 [file vaccines-13-00557-s001.zip › vaccines-3347433-supplementary/vaccines-3347433-supplementary/vaccines-3347433-supplementary.pdf]

Supplementary materials

Keywords and Search Strategy

Table S1. Keywords and search strategy

General database search strategy	
Population: pregnant Women	Maternal* OR Mother* OR Women OR Female OR Antenatal OR prenatal OR Pregnancy OR “Pregnant women” Or Gestation OR “Pregnant people” OR “During pregnancy” OR “Maternal health services”
Concept: vaccination	Vaccine* OR “Inactivated Vaccines” OR Inoculation OR “Maternal Vaccination” OR “Vaccination during pregnancy” OR Immunisation OR immunization OR Immunity
Concept: uptake/hesitancy	Uptake OR Acceptance OR Barrier* OR Hesitancy* OR Refusal OR Attitude OR Anxiety OR Fear OR Doubt
Context: Ireland	“Republic of Ireland” OR Ireland OR Irish OR “Northern Ireland” OR “All Ireland”
Lenus search strategy	Vaccination during pregnancy OR Maternal vaccination OR Maternal immunization AND Ireland<br>Filters: Year 2013 to 2023; only Articles

General database searches for each PCC (population, concept, and context) element were combined with AND with no limitations for year of publication or article type were used.

Example of final search strategy for PubMed

Search: (((Maternal* OR Mother* OR Women OR Female OR Antenatal OR prenatal OR Pregnancy OR "Pregnant women" Or Gestation OR "Pregnant people" OR "During pregnancy" OR "Maternal health services") AND (Vaccine* OR "Inactivated Vaccines" OR Inoculation OR "Maternal Vaccination" OR "Vaccination during pregnancy" OR Immunisation OR immunization OR Immunity)) AND (Uptake OR Acceptance OR Barrier* OR Hesitancy* OR Refusal OR Attitude OR Anxiety OR Fear OR Doubt)) AND ("Republic of Ireland" OR Ireland OR Irish OR "Northern Ireland" OR "All Ireland")

Supplementary materials

Table S2. List of Thomson's 5A dimensions and factors determining vaccine uptake

Dimension	Factors
**Access:** The ability of individuals to be reached by, or to reach, recommended vaccines.	• Convenience of access<br>• Vaccination location<br>• Contact with healthcare systems
**Affordability**: The ability of individuals to afford vaccination, both in terms of financial and non-financial costs (e.g., time).	• Financial incentives<br>• Time costs
**Awareness:** The degree, to which individuals have knowledge of the need for, and availability of, recommended vaccines and their objective benefits and risks.	• Knowledge of vaccines & vaccine schedule<br>• Availability of information<br>• Consideration of vaccination
**Acceptance:** - The degree to which individuals accept, question, or refuse vaccination.	
Disease	• Perceived severity<br>• Vulnerability to risk
Social context	• HCW influence<br>• Peer influence<br>• Social responsibility

Vaccine	• Attitude valence<br>• Perceived safety<br>• Perceived efficacy
Individual characteristics	• Health beliefs<br>• Past behaviour<br>• Trust<br>• Omission bias
**Activation:** the degree to which individuals are nudged towards vaccination uptake.	• Prompts & reminders<br>• Workplace policies

Supplementary results

Table S3. Summarised study characteristics of reviewed articles

	1	2	3	4	5	6	7	8	9	10	11	12		
	Lutejin 2011	Cleary 2014	Crosby 2016	O'Connell 2017	Barrett 2018	Hallisey 2018	Maisa 2018	O'Shea 2018	Ugezu 2018	Quattrochi 2019	Geoghegan 2021	Cuelmans 2021	Total number of papers	% of papers
Participants														
HCPs				x	x				x				3	25
pregnant women*		x	x		pp	x	x	pp	x	x	x	bf	10	83.3
Other	x												1	8.3

Vaccine type														
Influenza	x	x	x		x	x	x	x	x	x			9	75
Pertussis				x	x	x	x	x	x	x			7	
COVID											x	x	2	16.7
Study design/methods														
Quantitative		x	x		x	x			x	x		x	7	58.3
Qualitative							x	x					2	16.7
Mixed methods	x			x							x		3	25
Recruitment site														
Maternity hospital					x			x			x		3	25
Antenatal clinic/class			x						x				2	16.7
GP practice				x		x							2	16.7
Community (in person or online)							x			x		x	3	25
Location														
Multinational	x											x	2	16.7
Dublin			x		x			x			x		4	33.3
East of Ireland						x[#]			x				2	16.7
West of Ireland				x									1	8.3
Republic of Ireland										x			1	8.3
Northern Ireland (Belfast)							x						1	8.3

* pp: post-partum; bf: breastfeeding

[#] And South-East of Ireland

Supplementary results

Table S4. Summary of reviewed articles

Author/Year	Title	Study site & location in Ireland	Aim/purpose	Study methodology/design; population; sample	Vaccination type	Factors associated with (or related to) vaccination uptake or hesitancy	5A dimensions
Luteijn et al. 2011	Differences in pandemic influenza vaccination policies for pregnant women in Europe.	Europe: 24 countries	To describe the vaccination policies in European countries to enable discussion and reflection on the differences between pandemic vaccination policies and vaccination coverage of pregnant women between European countries.	Mixed methods survey of European Surveillance of Congenital Anomalies register leaders concerning antiviral use, vaccination policy and offer/uptake by pregnant women and women of childbearing age; country respondents n=24.	Influenza; Celvapan (Baxter) and Pandemrix (Glaxo Smith Kline) H1N1	International vaccine policy variations; pregnant women prioritized; variations in gestational period recommendations; funding models; safety data.	Access
Cleary et al. 2014	2009 A/H1N1 influenza vaccination in pregnancy: uptake and pregnancy outcomes - a historical cohort study.	Maternity hospital - Coombe Women and Infants University Hospital, Dublin, Ireland.	To describe the uptake of 2009 A/H1N1 influenza vaccination among pregnant women and determine if vaccination was associated with adverse pregnancy outcomes.	Quantitative: retrospective cohort study of pregnant women; (n=6894).	Influenza	Age, socioeconomic group, nationality, marriage status, planned pregnancy, gestation on booking antenatal care, mode of funded obstetric care, smoking status. Observation of maternal and infant outcomes in vaccinated and unvaccinated groups, demonstrating	Access; Affordability Acceptance

						vaccine safety and disease protection.	
Crosby et al. 2016	Uptake of the influenza vaccination in pregnancy.	Antenatal clinic, National Maternity Hospital, Dublin, Ireland.	To determine the uptake of the annual influenza vaccine in a pregnant population and ascertain the reasons why some pregnant women did not receive it.	Quantitative: prospective cohort study of women attending the antenatal clinic; (n=504).	Influenza	Safety concerns, misinformation, healthcare provider recommendations.	Affordability<br>Awareness<br>Activation
O'Connell et al.<br>2017	Antenatal Pertussis Vaccination: Why are General Practitioners Reluctant? A Mixed Methods Study Setting.	GP clinics in west of Ireland, Co Galway, Co. Mayo, and Co. Roscommon, Ireland.	To evaluate the current practices and attitudes of GPs in the West of Ireland with regard to antenatal pertussis vaccination.	Mixed methods: embedded mixed methods questionnaire with GPs (n=109); Invitation and return by post & self-administered.	Pertussis	GP attitudes and perceptions; concerns about vaccine safety and efficacy; GP recommendations to improve uptake.	Affordability<br>Acceptance<br>Awareness
Barrett et al. 2018	Influenza vaccination in pregnancy: vaccine uptake, maternal and healthcare providers'	Maternity hospital<br>- Rotunda Hospital, a tertiary referral maternity hospital in	To determine uptake of influenza vaccination during pregnancy; knowledge, attitudes, and concerns of postnatal women;	Quantitative: Paper-based survey of postnatal women attending the maternity hospital (n=198); online survey of HCPs (total n=1180: GPs (n=432)	Influenza & pertussis	Maternal knowledge; attitudes, beliefs, convenience, healthcare provider recommendations, HCPs knowledge and attitudes.	Access<br>Affordability<br>Acceptance<br>Awareness<br>Activation

	knowledge and attitudes. A quantitative study	Dublin, Ireland	and knowledge and attitudes of healthcare professionals (HCPs) surrounding vaccination.	pharmacists (n=419), and hospital site clinical staff (n=29: consultant obstetricians, non-consultant doctors, and midwives).			
Hallissey et al. 2018	Factors that Influence Uptake of Vaccination in Pregnancy.	Two GP clinics - Keogh Practice, Waterford city & Ballyhale, Co Kilkenny.	[To] understand the influences and barriers surrounding vaccination uptake amongst pregnant patients.	Quantitative: prospective cohort study; self-administered questionnaire; pregnant women attending routine antenatal appointments (n=88).	Influenza; pertussis	Maternal knowledge, attitudes, beliefs, convenience, HCP recommendations, socioeconomic and cultural Factors.	Awareness Acceptance Activation
Maisa et al. 2018	Vaccination against pertussis and influenza in pregnancy: a qualitative study of barriers and facilitators.	Public spaces; Belfast, Northern Ireland, UK.	[To] learn about the views of pregnant women and identifying potential barriers to vaccination in pregnancy.	Qualitative: focus group discussions and interviews with pregnant women recruited on-street; data collection executed by market research company; phenomenological approach (n=16).	Influenza; pertussis	Safety concerns, lack of knowledge, HCP recommendations, education, access to information, discussion with HCP	Access Affordability Awareness Acceptance
O'Shea et al. 2018	To vaccinate or not to vaccinate? Women's perception of vaccination in pregnancy: a	Maternity hospital - Rotunda Hospital, Dublin, Ireland.	To explore women's perception of vaccination in pregnancy and thereby determine the reasons behind	Qualitative: semi-structured telephone interviews; women one-month post-partum (n=17); part of nested mixed-methods study; from the quantitative	Influenza; pertussis	Perceptions of risk, safety concerns, information needs; healthcare providers influence pregnant women's choice to vaccinate; lack of understanding regarding	Access Awareness Acceptance

	qualitative study.		such low vaccination rates.	component (Barrett et al 2018).		vaccine safety regarding pertussis vaccination.	
Ugezu and Essajee 2018	Exploring patients' awareness and healthcare professionals' knowledge and attitude to pertussis and influenza vaccination during the antenatal periods in Cavan Monaghan general hospital.	Regional hospital antenatal clinic - Regional hospital - Northeast Ireland.	To assess patients' awareness of pertussis and influenza vaccination as well as healthcare professionals' knowledge and attitude to pertussis and influenza vaccination during the antenatal periods in Cavan Monaghan General Hospital.	Quantitative: prospective cohort study; survey of healthcare professionals (non-consultant hospital doctors and midwives) (n=50); survey of pregnant women attending routine antenatal care (n=113)	Influenza; pertussis	HCP Knowledge gaps, healthcare provider attitudes and practices; HCP recommendations; pregnant women's awareness; communication.	Awareness Acceptance Activation
Quattrocchi et al. 2019	Determinants of influenza and pertussis vaccine uptake in pregnant women in Ireland: A cross-sectional survey in 2017/18 influenza season.	Republic of Ireland, all provinces.	To estimate uptake of seasonal influ-enza and pertussis vaccines during pregnancy, and to identify factors associated with uptake of these vaccines during the 2017/18 influenza season in Ireland.	Quantitative: cross-sectional household omnibus* face-to-face survey; pregnant women (n=241) *omnibus survey - survey questions included in a larger survey conducted by a market research company.	Influenza; pertussis	HCP recommendations; vaccine knowledge and awareness; awareness of public health campaigns; socioeconomic status; geographic location; reasons for getting or not getting vaccinated; health beliefs.	Access Awareness Acceptance Activation

Geoghegan et al. 2021	"This choice does not just affect me." Attitudes of pregnant women toward COVID-19 vaccines: a mixed-methods study.	Maternity hospital - Rotunda Hospital, Dublin, Ireland	To investigate the attitudes of pregnant women toward COVID-19 vaccines, so that women may be supported to make the best decision for their individual risk profiles.	Mixed methods; online survey; pregnant women (n=300) recruited while attending the hospital.	COVID-19	Older age; later gestational stage; private/semi-private clinic; vaccine safety concerns for unborn; safety research - inclusion of pregnant women in clinical trials; demonstrating safety through data; introduction of a new vaccine, fear and anxiety, concern about long term effects; trust in HCP recommendations; attitudes, beliefs, information sources, decision-making factors.	Affordability<br>Awareness<br>Acceptance
Ceulemans et al. 2021	Vaccine Willingness and Impact of the COVID-19 Pandemic on Women's Perinatal Experiences and Practices -A Multinational, Cross-Sectional Study of the Pandemic.	6 European countries: Ireland, Norway, Switzerland, Netherlands, United Kingdom, and Belgium.	To explore beliefs about the coronavirus and COVID-19 vaccine willingness and to assess the impact of the pandemic on perinatal experiences and practices.	Quantitative: multinational, cross-sectional, web-based survey; recruitment via promotion of study on social media and websites visited by pregnant women; total participants n=16,063: pregnant women (n= 6661), breastfeeding women (n=9402). 10% of respondents in Ireland.	COVID-19	Vaccine confidence, risk perception, access and availability, information and communication, social influence, prioritization and eligibility and, personal and cultural beliefs; perceived impact and risks; health disparities and inequalities; perinatal experiences during pandemic.	Affordability<br>Acceptance

Table S5. Factors associated with maternal vaccination uptake/hesitancy.

		1	2	3	4	5	6	7	8	9	10	11	12		
● Evidence based relationship to uptake<br>○ Related to topic of vaccination uptake but lacking evidence of association with vaccine status		Lutejin 2011	Cleary 2014	Crosby 2016	O'Connell 2017	Barrett 2018	Hallisey 2018	Maisa 2018	O'Shea 2018	Ugezu 2018	Quattrochi 2019	Geoghegan 2021	Cuelmans 2021	Total number of studies	(%)
**Access**			x			x		x	x		x			5	41.7
Convenience of access															
	– *Women have responsibility for making vaccine appointment*							○						1	8.3
	– *Accessible through multiple HCPs or healthcare settings*		○			○			○		○			4	33.3
Vaccination location	– *Resident in Leinster*										●			1	8.3
Contact with healthcare systems	– *Booking antenatal care at gestation >20 weeks*		●											1	8.3
**Affordability**			x	x	x	x		x				x	x	7	58.3
Financial incentives	– *Private or publicly funded obstetric care*		●	●		●						●		4	33.3
	– *Variation in GP reimbursement policy for different vaccines*				○									1	8.3
Time costs	– *Having time to receive the vaccine*							○						1	8.3
	– *Perceived time of HCP to discuss vaccination*							○						1	8.3

Table S5. continued

**Awareness**				x	x	x	x	x	x	x	x	x		9	75.0
Knowledge of vaccines & vaccine schedule	– *Maternal lack of awareness & knowledge of recommended vaccines during pregnancy*			●			○	○	○	○				5	41.7
	– *Maternal lack of understanding that vaccination protects the foetus*							○						1	8.3
	– *Maternal lack of knowledge on safety of specific vaccines*								○	●				2	16.7
	– *Maternal perception that some vaccines are more important than others*								○					1	8.3
	– *HCP lack of knowledge of recommended vaccine schedules in pregnancy*				○	○				●				3	25.0
Availability of information	– *HCP lack of discussion of vaccine with pregnant women*							○						1	8.3
	– *Lack of information & support from national health service or obstetric teams for GPs on maternal vaccination*				○									1	8.3
	– *Lack of information from HCP for pregnant women*									●				1	8.3
	– *Seeking information outside of healthcare settings*					○		○	●		○	○		5	41.7
Consideration of vaccination	– *Forgetting to get vaccinated*			●										1	8.3

Table S5. Continued

**Acceptance**				x		x	x	x	x	x	x	x	x	x	10	83.3
Disease																
	Perceived severity	– *Perceived severity for pregnant women*					○	○							2	16.7
		– *Perceived severity for foetus/newborn*				○	○	○						○	3	25.0
		– *Knowledge that severe infection during pregnancy can affect foetal development*												○	1	8.3
		– *Maternal perception some illnesses are mild (influenza)*								○					1	8.3
	Vulnerability to risk	– *Awareness & knowledge of vulnerability to risks from infection during pregnancy*					○							○	3	25.0
		– *Evidence of vulnerability - testing positive for COVID 19*												●	1	8.3
Social context																
	HCW influence	– *Positive influence of HCP*					●			●	●				3	25.0
		– *HCP recommendation*						○	○	○	○	○	○		6	50.0
		– *Receiving information from a HCP*					●				●				2	16.7
		– *HCP offer of vaccine*					●								1	8.3
		– *HCP discussion AND offer of vaccine*									●				1	8.3
		– *HCPs sociodemographic characteristic differences in recommending vaccination*					○								1	8.3
		– *Potential of midwife recommendations*											○		1	8.3
		– *Lack of clarity in whose role it is to advise and recommend vaccines*						○					○		2	16.7
		– *HCP reluctance to recommend*				○									1	8.3
		– *HCPs fear of litigation as result of adverse events in pregnancy*				○									1	8.3

Table S5. Continued (Acceptance)

HCW influence (continued)	– *HCP see it as woman's prerogative to raise discussion*				○									1	8.3
Peer influence	– *Partner influence*							○						1	8.3
	– *Family and friend influence*							○	○	○				3	25.0
Social responsibility	– *Woman's sense of responsibility for protecting baby*							○				○		2	16.7
	– *HCP sense of responsibility to recommend the vaccine*					●			●					2	16.7
Vaccine															0.0
Attitude valence	– *Vaccination in previous pregnancy*					●					●			2	16.7
Perceived safety	– *Safety concerns (maternal/HCP)*				●	●	○	●	●	●	○	○		8	66.7
	– *Concern about perceived link between vaccines and long-term conditions*														
Perceived efficacy	– *Perception people being infected after vaccination is evidence of poor efficacy*										○			1	8.3
Individual characteristics															
Pregnancy	– *Being pregnant*										○	○		2	16.7
	– *Planned/unplanned pregnancy*		●											1	8.3
	– *Number of pregnancies*												●	1	8.3
	– *Number of births*			○									●	2	16.7
	– *Breast feeding*												●	1	8.3
	– *Gestational stage*											●		1	8.3
Health beliefs	– *Belief that medication should be avoided in pregnancy (HCP and maternal)*				○			○						2	16.7
	– *Belief there are too many vaccines*							○						1	8.3

Table S5. Continued (Acceptance)

Health beliefs (continued)	– *Belief natural immunity is best*							○						1	8.3
Past behaviour	– *Being vaccinated in a previous pregnancy (evidence of positive attitude valence)*					●	?								0.0
Trust	– *Trust in HCP knowledge or advice*						○	○	○			○		4	33.3
	– *Difficulty building trust due to variation in HCP during course of pregnancy*							○						1	8.3
Omission bias															
**Activation**					x	x					x			3	25.0
Prompts & reminders	– *Letters to GPs and PH nurses to encourage vaccine recommendation following outbreak*										○			1	8.3
	– *Maternal awareness of Public Health Vaccination campaigns*										●			1	8.3
	– *HCPs recommend inclusion of a reminder in the National Maternity Notes*				○									1	8.3
	– *HCPs recommend national vaccination media campaign*				○									1	8.3
Workplace policies	– *Low HCPs vaccination rates despite workplace guidelines for vaccination*					○				○				2	16.7

HCP – Healthcare providers; GP – general practitioners; PH – public health (nurse).
